# Radial Profile-Based Quantification of Centrosomal Proteins

**DOI:** 10.21769/BioProtoc.5638

**Published:** 2026-03-20

**Authors:** Alan Wainman

**Affiliations:** Sir William Dunn School of Pathology, University of Oxford, Oxford, UK

**Keywords:** *Drosophila* embryo, Centrosome dynamics, Radial profile analysis, Quantitative fluorescence imaging, FRAP, 3D-SIM, Super-resolution microscopy, Pericentriolar material, Centriole

## Abstract

Centrosomes are dynamic organelles critical for mitotic spindle assembly and cilia formation. Here, I describe a protocol for quantifying relative centrosomal protein abundance in *Drosophila melanogaster* embryos using radial profile analysis of fluorescence intensity. The method involves embryo collection, manual dechorionation, mounting for live imaging, confocal microscopy, and subsequent image analysis. Radial profiling allows quantification of relative protein abundance together with its spatial distribution at the centrosome, providing either relative or normalized intensity profiles. I then outline how this approach can be integrated with complementary techniques such as fluorescence recovery after photobleaching (FRAP) and super-resolution imaging, in this case, three-dimensional structured illumination microscopy (3D-SIM). Combining radial fluorescence profiling with these imaging modalities enables high-resolution, quantitative analysis of dynamic centrosome assembly in a genetically tractable system.

Key features

• Manual embryo dechorionation and heptane glue mounting enable reliable preparation for high-quality live imaging.

• Radial profile analysis provides quantitative measurement of centrosomal protein abundance and spatial distribution.

• The protocol is compatible with both confocal and 3D-SIM super-resolution microscopy, offering complementary spatial and temporal resolution.

**This protocol is used in:** Sci Adv (2025), DOI: 10.1126/sciadv.adr5744

## Graphical overview


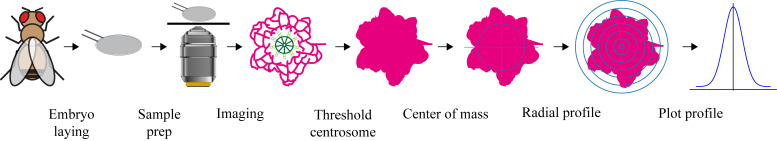


**Workflow for centrosome radial profiling.** *Drosophila* embryos are imaged, centrosomes segmented, centroids determined, and radial fluorescence intensity profiles generated for quantitative analysis.

## Background

The centrosome is a cellular organelle composed of a pair of orthogonally arranged, cylindrical centrioles surrounded by an amorphous mesh of various proteins, called pericentriolar material (PCM). Centrosomes are important for many cellular functions, most notably spindle pole organization during mitosis and ciliogenesis in quiescent cells [1–3].

The centrosome is not a static organelle but rather a dynamic structure that varies in both composition and organization throughout the cell cycle [4–7]. Two notable events during the cell cycle are (i) the growth of a daughter centriole (on the side of the preexisting mother centriole) during S phase and (ii) the dramatic increase in PCM volume on the entry into mitosis. Monitoring these events live in an in vivo system is essential to fully capture their dynamics, which can range from seconds (e.g., mitotic kinase turnover) to minutes (e.g., the dynamics of pericentriolar material scaffold proteins such as Cnn and Spd-2) [8]. Defining how and where centrosomal proteins are recruited and quantifying their abundance is fundamental to modeling centrosome formation. Quantification through radial profile analysis can be presented either with intensity levels normalized to the average peak value of the wild-type protein, allowing comparison of protein abundance between genetic backgrounds, or with intensity levels normalized so that the peak intensity is set to 1, allowing comparisons of protein localization irrespective of protein abundance at the centrosome.

Radial profiling of centrosomal fluorescence intensity, using an analysis method similar to the one described here, has been routinely employed to investigate centrosome organization in *Caenorhabditis elegans* [9,10]. In the present work, we describe its implementation in *Drosophila*, although the same analytical approach is readily applicable to centrosomes in other model organisms and experimental systems.

The syncytial *Drosophila* embryo is a powerful system for quantifying centrosome assembly dynamics [11]. During the first 13 nuclear cycles, nuclei divide in a shared cytoplasm without cytokinesis. In cycles 10–13, centrosomes localize at the embryonic cortex, where they are ideally positioned for imaging near the coverslip [12,13]. These divisions are nearly synchronous, allowing multiple events to be quantified in parallel; combined with the large number of centrosomes undergoing rapid processes, this enables rigorous statistical analysis [14]. Together with genetic tractability, this enables integration with mathematical modeling, providing fundamental insights into centrosome biology [15,16].

Centrosomes in the fly embryo have been imaged using conventional microscopy for many years [17]. Recently, two imaging techniques, fluorescence recovery after photobleaching (FRAP) and three-dimensional structured-illumination microscopy (3D-SIM), have been applied to this system to examine the dynamics of centrosome assembly in greater detail. FRAP allows the dynamic incorporation of centrosomal proteins to be investigated [8,18,19]. In this technique, fluorescently tagged proteins within the centrosome are bleached with a high-power laser. The subsequent replacement of bleached molecules at the centrosome with unbleached molecules from the cytoplasm provides details on incorporation location and dynamics. 3D-SIM extends the resolution of imaging beyond that defined by Abbe’s law of diffraction [20,21]. In this technique, the sample is illuminated with patterned light that interferes with the sub-resolution structures within the sample. The Moiré interference pattern generated enables the sub-diffraction information to be algorithmically decoded and reconstructed [22,23]. This enhanced resolution has revealed finer details of centrosomal protein localization [24–27]. Indeed, FRAP and 3D-SIM can be combined to provide the localization of incorporating molecules at a higher resolution [8,28,29].

Here, I provide guidance on how to use radial profile analysis to quantify centrosome protein abundance in the *Drosophila* embryo imaged by standard confocal microscopy and then demonstrate its application in combination with FRAP and 3D-SIM. I illustrate the various approaches with previously unpublished original data.

## Materials and reagents

**Biological materials**

The imaging approaches described in this protocol assume that a fly line is used where the protein(s) of interest are fluorescently tagged. Wherever available, lines expressing fluorescently tagged proteins under the control of the endogenous promoter in a mutant background are selected, as these provide expression levels close to endogenous, confirmed by western blotting. The fluorescent protein selected depends on experimental requirements. For two-color experiments, eGFP, NeonGreen [30], or StayGold [31] (the latter generally more photostable) are typically combined with mCherry, a relatively bright and stable red fluorophore [32,33].

**Reagents**

1. Instant dried yeast (Fermipan red)

2. Bacteriological agar (VWR, catalog number: 84609)

3. Sucrose (Sigma-Aldrich, catalog number: RDD023)

4. Apple or cranberry juice

5. Heptane (Sigma-Aldrich, catalog number: 246654)

6. Voltalef H10S PCTFE oil ARKEMA (Samaro, catalog number: AK6208)

7. Immersion oil for 3D-SIM imaging on OMX-Blaze, DV Immersion Oil Kit containing refractive index oil 1.514 (GE Healthcare, catalog number: 29163068)

8. Immersion oil with a refractive index of 1.518 for spinning disc confocal (ImmersolT 518 F, Carl Zeiss, catalog number: 433802-9010-000)

**Solutions**

1. Yeast paste (see Recipes)

2. Fruit juice agar plates (see Recipes)

3. Heptane glue (see Recipes)

**Recipes**

**1. Yeast paste**

Mix instant dried yeast with water (approximately 10 g of yeast in 12 mL of water) in a 100 mL beaker to achieve a thick, creamy consistency.

**2. Fruit juice agar plates**

a. Boil 9 g of agar in 300 mL of distilled water and, separately, 10 g of sucrose in 100 mL of apple juice (or cranberry juice).

b. Mix together the agar and sugar solutions once fully dissolved.

c. Pour the mix into Petri dishes of a size designed to fit snugly into the bottom of the fly collection chamber (typically a diameter of 50 mm and height of 20.3 mm) to a depth of ~15 mm. Leave to cool and dry at room temperature for ~1 h before use. Plates can be stored at 4 °C for 2–3 days. Before use, add a button-sized drop of yeast paste to each plate and leave to dry. The yeast paste needs to dry to prevent the flies from getting stuck in the liquid yeast.

**3. Heptane glue**

a. Fill a 50 mL conical tube with double-sided sticky tape (remove the release liner and tightly pack approximately 1 m of tape into the tube) and add 20 mL of heptane. Leave the tube rotating overnight.

b. Remove the tape and centrifuge the heptane glue at 15,700 RCF to remove solids. The glue can be kept indefinitely at RT in an Eppendorf tube sealed with Parafilm.

**Laboratory supplies**

1. Petri dishes, 50 mm diameter (Fisher Scientific, catalog number: 10655821)

2. Glass-bottom 35 mm dishes (MatTek, catalog number: P35G-1.5-14-C)

3. Paintbrush (size 3)

4. Fine forceps (Dumont No. 5)

5. Microscope slides (Appleton Woods, catalog number: MS529)

6. Double-sided sticky tape (Scotch 665 Double-Sided Tape)

*Note: The adhesion of some brands of double-sided sticky tape is too strong to dechorionate the embryos successfully. Scotch tape is particularly well-suited for dechorionation.*

7. Sticky tape (single-sided) for holding the fruit juice agar plate onto the fly collection chamber (Starlabs ID Tape 55 m long × 19.0 mm wide, catalog number: E9055-1913)

## Equipment

1. Fly collection chamber: This is typically a Perspex cylinder with one end sealed with a fine wire mesh (so that flies can breathe but not escape) (Flystuff Inc., catalog numbers: 59-100 or 59-101)

2. Humidified incubator at 25 °C in a reverse day light cycle to optimize the embryo collection during standard laboratory working hours

3. Binocular dissecting microscope (Nikon, model: SMZ800)

4. Rotator mixer (Stuart, model: SB3)

5. Confocal microscope with FRAP unit

*Note: The example shown in Figure 3A was acquired using a Perkin Elmer Spinning disc system running Volocity 6.3 software mounted on a Zeiss Axiovert microscope with a 63×/1.4 NA oil immersion objective lens (60×/1.3 NA Silicone immersion oil lenses are also suitable). This system is equipped with a Hamamatsu Orca ER CCD camera and 488 and 568 nm laser lines. Comparable FRAP experiments can be performed on many other confocal systems.*

6. 3D-SIM microscope with *blaze* unit

*Note: The example shown in Figure 4A–E was acquired using a DeltaVision OMX V3 Blaze microscope (GE Healthcare), fitted with a 60×/1.42 NA UPlanSApo oil immersion objective lens. This system is fitted with PCO Edge 5.5 sCMOS cameras and 488 and 593 nm laser lines. Other SIM systems are suitable for similar experiments.*

## Software and datasets

1. Volocity (Perkin Elmer)

2. SoftWoRx (GE Healthcare)

3. ImageJ/FIJI (http://fiji.sc/) [34]

4. SIMcheck plugin (http://downloads.micron.ox.ac.uk/fiji_update/SIMcheck/) [35]

5. Radial Profile plugin (https://imagej.net/ij/plugins/radial-profile.html)

## Procedure

**A. *Drosophila* embryo collection and sample preparation**

**Figure 1.**
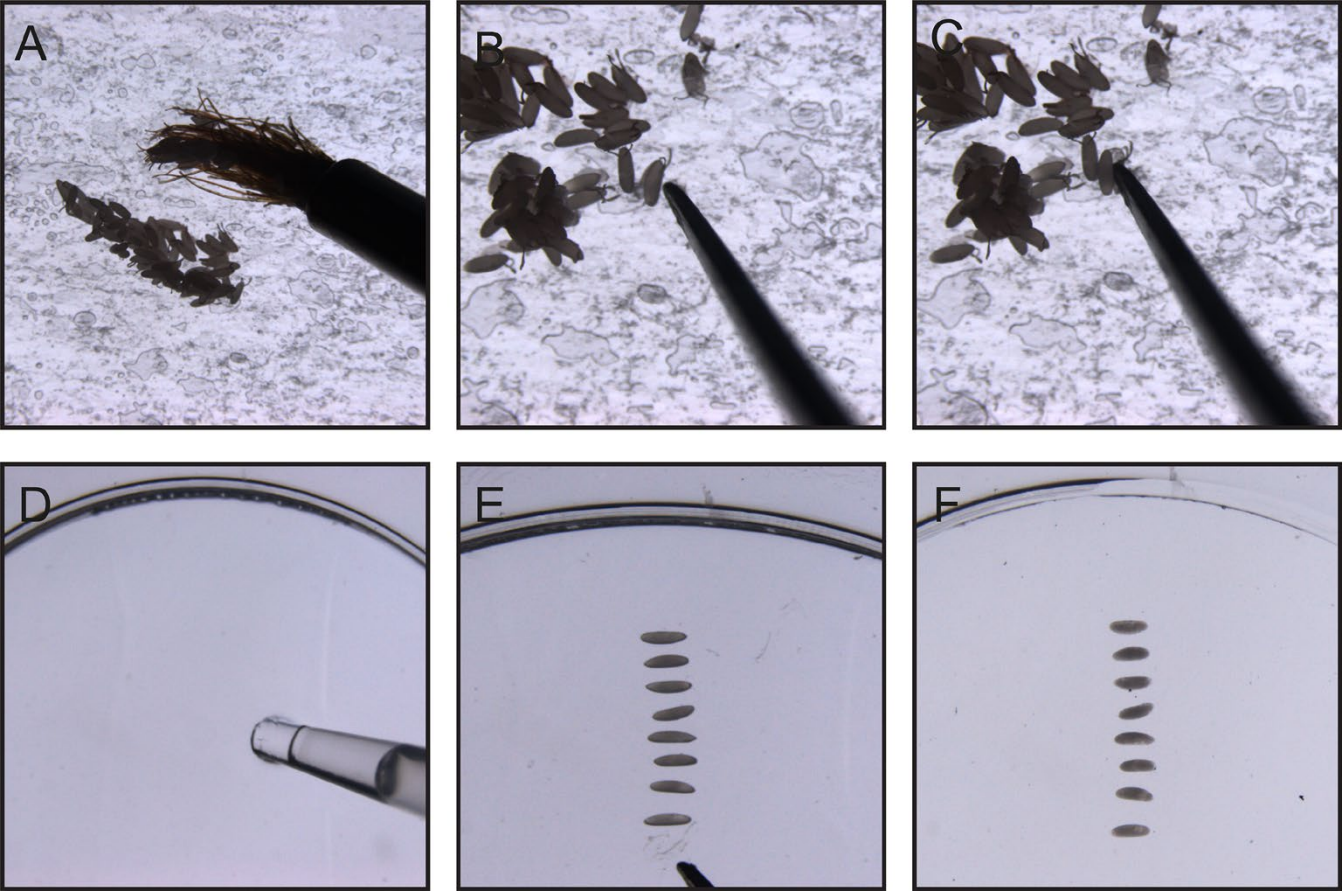
Manual dechorionation of *Drosophila* embryos. (A) (Step A8) Using a paintbrush, transfer embryos onto double-sided tape affixed to a glass slide. (B) (Step A9) Gently tap each embryo with the arm of a forceps to crack the chorion while avoiding damage to the embryo. (C) (Step A10) Carefully remove the embryo from the opened chorion and pick it up using the forceps arm. (D) (Step A11) Using a pipette tip, apply a thin, even layer of glue to the glass bottom of a MatTek dish. (E) (Step A12) Position the dechorionated embryo onto the glued surface. (F) (Step A13) Cover the mounted embryos with Voltalef oil to prevent desiccation.

Imaging the syncytial *Drosophila* embryo requires removal of the chorion (outer shell), as it is auto-fluorescent. This can either be done manually or using 60% bleach (see [36] for details). For the imaging experiments described here, only a small number of embryos are needed; therefore, manual dechorionation is used, as outlined below. Steps A8–13 are depicted in Figure 1.

1. Place flies in a cage.

2. Close the cage by placing a yeasted fruit juice agar plate at the open end and seal it with tape.

3. Turn upside down (plate side down) and place in a 25 °C incubator.

4. For the next 48 h, replace yeasted plates every 3 h during working hours (typically, 4 changes a day). Plates should be at 25 °C. This ensures females remain well fed and increases embryo laying. Changing plates outside core working hours provides minimal additional benefit and is excessive for routine experiments.

5. On the third day, change the yeasted plate and allow flies to lay for 1 h. Discard this first collection, as females tend to retain embryos overnight (a phenomenon known as facultative ovoviviparity [36,37]), and these may be past the optimal stage for imaging.

6. Collect embryos from the next yeasted plate after a further 1 h. An hour-long collection ensures that, by the time preparations are complete, centrosomes in most embryos are located at the cortex.

7. Age embryos for a further 30 min at 25 °C.

8. Transfer embryos from the plate to double-sided sticky tape stuck on a microscope slide using a paintbrush.

9. Under a dissecting microscope (at 4–7× magnification), gently tap the embryo with forceps to *crack open* the chorion (the outermost layer of the embryo).

10. Pick up the embryo by simply touching it with the end of one forceps arm. The embryo will easily stick to the forceps, leaving the chorion behind on the tape.

11. Add a thin layer of the heptane glue to the coverslip (number 1.5 thickness) of a 35 mm glass-bottom dish by dipping a 20–200 μL pipette tip in the heptane glue and letting it enter the tip by capillary action. Use the tip to draw a line of the heptane glue onto the glass-bottom dish. The heptane evaporates rapidly, leaving a thin layer of glue on the glass. Prepared dishes should be kept at room temperature and only made in quantities that will be used within the same day.

12. Place embryos on the glued surface of a 35 mm MatTek dish. You should endeavor to line up the embryos in a vertical line, approximately equidistantly, as this will make it easier to move from one embryo to the other when imaging. The process of removing the chorion and placing the dechorionated embryos in the dish should ideally take no longer than 15 minutes; 10–15 embryos are generally aligned per dish. Once removed from the chorion, the embryos will start to desiccate; ideally, embryos in the same line should be uniformly desiccated.

13. Cover the embryos in Voltalef oil so they are just submerged. Voltalef permits air diffusion, allowing the embryos to continue developing while preventing further desiccation. This oil has a refractive index similar to that of the immersion oil, the glass of MatTek dish, and the cytosol of the embryo.

**B. Imaging on a confocal microscope**

**Figure 2.**
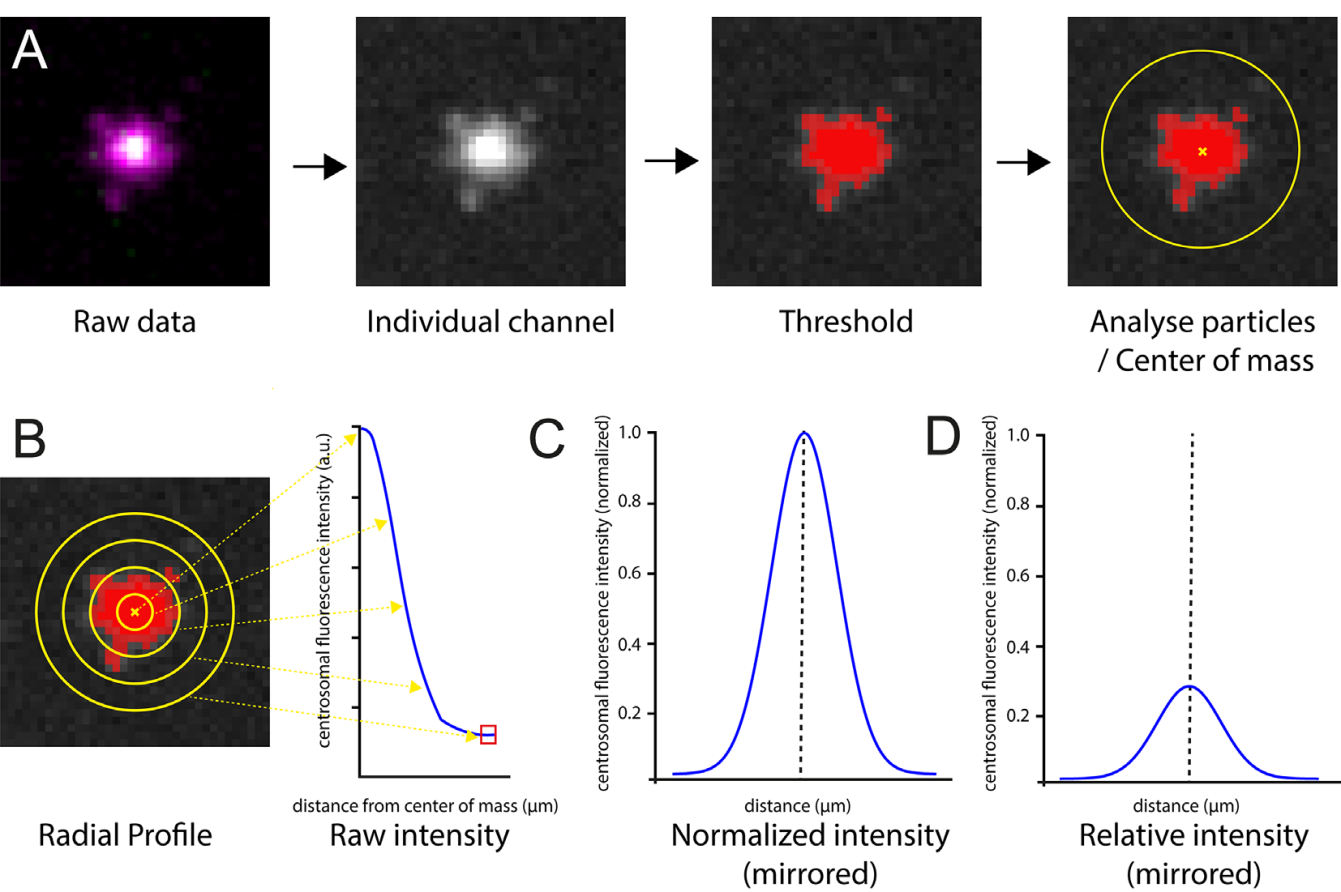
Workflow for generating centrosomal fluorescence intensity profiles. (A) In brief, raw images are acquired on a spinning disk confocal microscope and processed in ImageJ (in this example, a PCM marker Cnn is shown in magenta and centriole marker Asl in green). The channels are separated, the scale of the image is increased 5-fold, a threshold is applied to identify the centrosome signal above background, and the center of mass is identified using *Analyze particles*. (B) Radial profiles are then generated using the identified centroid. The background values are subtracted (represented by the red box), and values are mirrored. Profiles are then (C) normalized to the maximum within their specific dataset, illustrating the spatial distribution of the protein independent of abundance, or (D) normalized to a wild-type maximum intensity, allowing comparison between genetic backgrounds.

**Figure 3.**
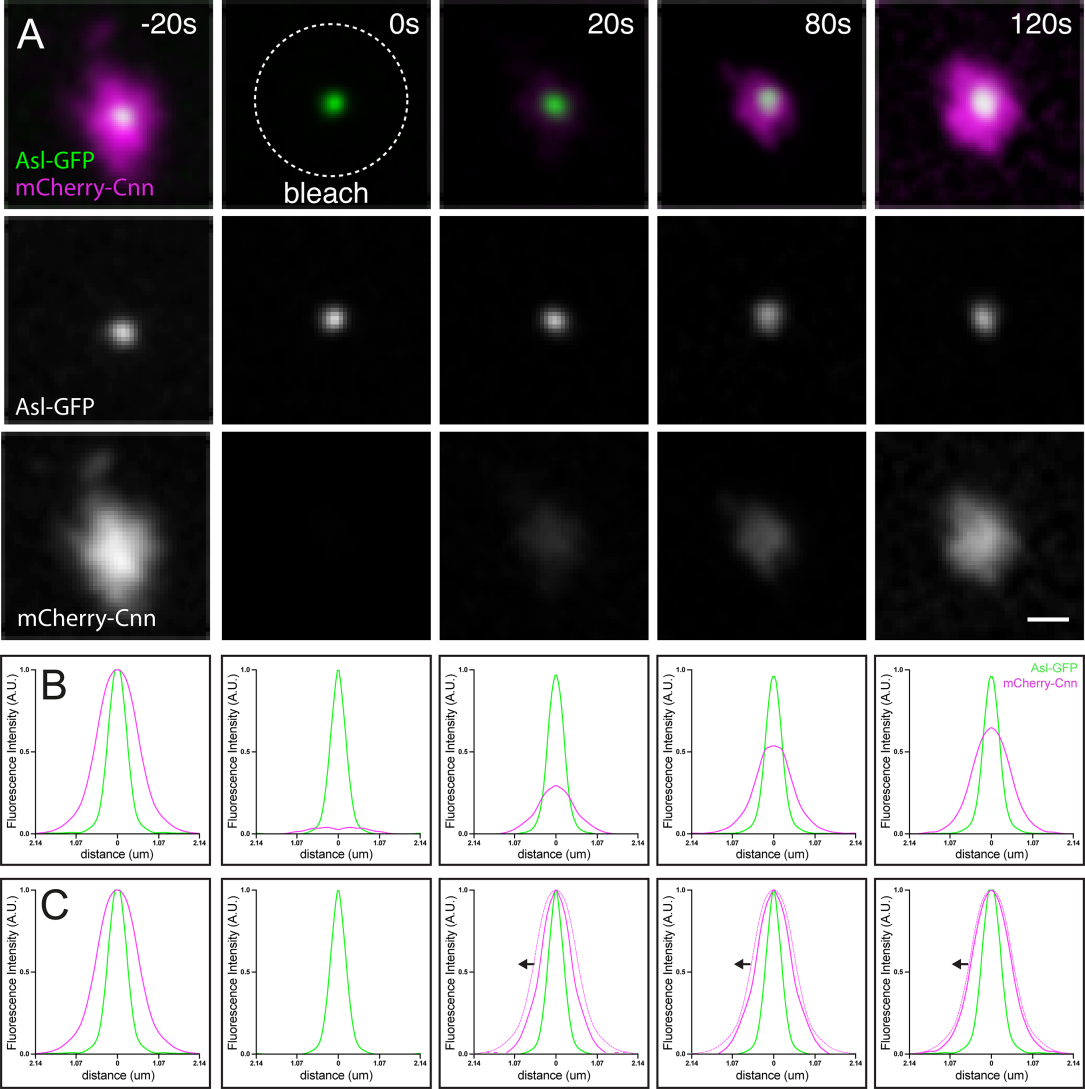
Combining radial profiling with fluorescence recovery after photobleaching (FRAP). (A) FRAP of mCherry-Cnn at centrosomes. Images show fluorescence before bleaching (-20 s), immediately after bleaching (0 s), and during recovery. Centrioles are marked with Asl-GFP. Scale bar: 1 μm (B) Radial profile of Asl-GFP and mCherry-Cnn for each time point. The values are normalized to the pre-bleach maximum intensity, allowing comparison of intensities between time points. (C) Radial profile of Asl-GFP and mCherry-Cnn for each time point. The values are normalized to the maximum intensity at each time point, allowing comparison of the spatial distribution of newly incorporated mCherry-Cnn molecules (arrows show inside-to-out spreading of Cnn signal during recovery [8]; thin magenta line shows pre-bleached profile for context; “bleach” panel shows Asl-GFP centriole signal only.

1. Place the dish on the microscope. A typical setup uses a 63× 1.4 NA objective mounted on a spinning disc confocal fitted with 488 nm (200 μW at sample) and 568 nm (300 μW at sample) laser lines.

2. Move the microscope stage to find an embryo within cycle 10–12, in which a large number of centrosomes will localize close to the cortex [12].

3. Find the middle of the centrosomes in the z-axis using the microscope focus. This is usually the height with the brightest fluorescence. At this developmental stage, all centrosomes in the field of view will be at approximately the same distance from the cortex. Set the z-stack acquisition such that 1) the step size provides Nyquist or near-Nyquist sampling, 2) the whole centrosome is captured, and 3) the brightest plane of the centrosome is positioned roughly in the center of the z-stack. In Figures 2A and 3A, a stack of 7 images at 0.5 μm intervals is shown with a pixel size of 0.219 μm × 0.219 μm.

4. Capture channels sequentially, capturing the entire stack first before changing channels (if confocal has only one camera).

5. Set your imaging conditions to minimize exposure time while guaranteeing a good signal-to-noise/background ratio.

*Note: It is essential to avoid camera saturation to ensure accurate quantification.*

6. To ensure reproducibility across experiments, measure the laser power using a slide light meter (Thorlabs). Adjust software settings so that the same laser output at the objective is maintained for all experiments (rather than simply keeping software laser power settings constant).

**C. Image analysis**

Here, I describe how to carry out radial profile image analysis to quantify the abundance of proteins at the centrosome. The image analysis workflow is shown in Figure 2. A radial fluorescence intensity profile measures the average fluorescence intensity at successive distances from the centrosome center, calculated along a circular region of interest (Figure 2B). This approach also provides information on the spatial distribution of protein localization at the centrosome.

1. Open the image in FIJI/ImageJ [34].

2. For two-channel images, split the channels (*Image* > *Color* > *Split Channels*).

3. Scale the image so that each pixel is divided into 25 (5 × 5) pixels (*Image* > *Scale*, use *Bilinear interpolation* option). Although not essential, this step will help smooth the final radial profile graph, making it more appealing for presentation.

4. Remove the pixel scaling (*Analyze* > *Set scale* > *Click to remove scale*). The radial profile plugin is configured to use pixels as a unit of length.

5. Convert the image to 8-bit (*Image* > *Type* > *8-bit*). Although not essential, as the procedure should also work with images of other bit depths, this step aids comparison of data recorded on cameras with different bit depths.

6. Segment the image using the threshold function to identify the centrosomes (*Image* > *Adjust* > *Threshold – Otsu method*). Set the levels by eye to ensure that the entire region of the centrosome is segmented into one object.

7. Identify the z-slice where the centrosomes are in best focus by finding the z-slice where they have the highest intensity values.

8. Draw a region of interest (ROI) around a centrosome. Set ROI to a circular shape and resize so that the centrosome to be analyzed just falls into the area defined.

9. Set measurements to include *Center of Mass (Analyze* > *Set Measurements*).

10. Determine the center of mass of the centrosome using the *Analyze Particle* function (*Analyze* > *Analyze Particles*). This will provide the coordinates of the centrosome center.

11. Use the ImageJ radial profile plugin (https://imagej.net/ij/plugins/radial-profile.html) by adding in coordinates from step C9 (*Plugins* > *Radial Profile*; Figure 2B). The radius of the radial profile should extend beyond the signal for the protein of interest. The same profile size must then be applied to all centrosomes within an experiment.

12. Copy values into an Excel spreadsheet.

13. In Excel, subtract the average cytosolic signal from each profile (usually the last five values given by the radial profile plugin, corresponding to the outermost pixels of the radial profile in the cytosol surrounding the centrosome).

14. Normalize intensity values. Two approaches can be used:

a. To compare spatial localization independent of abundance, normalize profiles so that the peak intensity is set to 1 (Figure 2C).

b. To compare the relative abundance of a protein at the centrosome, normalize to the average peak value of the wild-type protein (Figure 2D). As the abundance of most, if not all, centriole and centrosome proteins fluctuates throughout the cell cycle, ensure that protein levels are compared between images taken at the same cell cycle stage. (The peak value in each profile from centrosomes imaged under wild-type conditions is determined in Excel using the =MAX() function for the corresponding column of values. The average of these peak values across all individual centrosome profiles is then calculated in Excel.)

15. Mirror the profiles in Excel to generate a full symmetric centrosomal profile.

16. Pixels are converted into distance in Excel using the known length of each pixel (with five values representing one pixel length due to the upscaling in step C3).

17. For each condition, calculate the average distribution from at least five centrosomes in each of seven or more embryos (a minimum of 35 measurements).

**D. FRAP imaging on a confocal microscope**

FRAP is used to assess the dynamic exchange of proteins between the centrosome and cytoplasm and to quantify the site of protein incorporation within the centrosome. Here, I describe how FRAP is performed on centrosomes using a spinning disc confocal microscope. In the example shown in Figure 3A, I use a fly stock where the protein of interest, the PCM protein Cnn, is tagged with mCherry, and a general centrosomal marker Asl is tagged with eGFP. A GFP marker is required to define the centrosome position when the mCherry marker is bleached.

1. Collect and prepare the embryos as described in section A.

2. Place embryos on the microscope and set imaging settings as described in section B.

3. Define a bleaching ROI to include a single centrosome using the software interface. The ROI should be only slightly larger than the fluorescent signal. If the ROI is too large, the recovery dynamic will be confounded by bleaching of surrounding cytosolic proteins.

4. Optimize the laser settings so that the mCherry signal is completely bleached while the GFP signal is unaffected. In the example shown in Figure 3A, the mCherry signal was fully bleached with 5 iterations of 100% 568 nm laser (~2 mW).

5. Optimize post-bleach acquisition timing according to the dynamics of the protein of interest. The faster the recovery of the fluorescent signal, the shorter the interval between acquisitions. In an ideal scenario, apply Nyquist sampling so that the frame rate is at least twice the frequency of the dynamics of interest. In practice, this interval must be carefully balanced against the need to minimize photobleaching during image acquisition. In the example shown in Figure 3A, mCherry-Cnn recovered measurably within 30 s, so a 20 s acquisition interval was used.

6. Start the experiment by acquiring at least two pre-bleach frames. Bleach several centrosomes in the field of view by setting several ROIs and acquire the necessary post-bleach time points. In Figure 3A, meaningful data were obtained from 15 or more centrosomes from at least 5 embryos.

7. Analyze each time point as described in section C. In Figure 3B, profiles are normalized compared to the pre-bleached mCherry-Cnn signal, so that the peak intensity is set to 1, giving the relative abundance of the newly incorporated mCherry-Cnn molecules. In Figure 3C, profiles are normalized compared to the maximum mCherry-Cnn signal at each time point, so that the peak intensity is set to 1, giving the spatial distribution of the newly incorporated mCherry-Cnn molecules.

**E. 3D-SIM Imaging**

**Figure 4.**
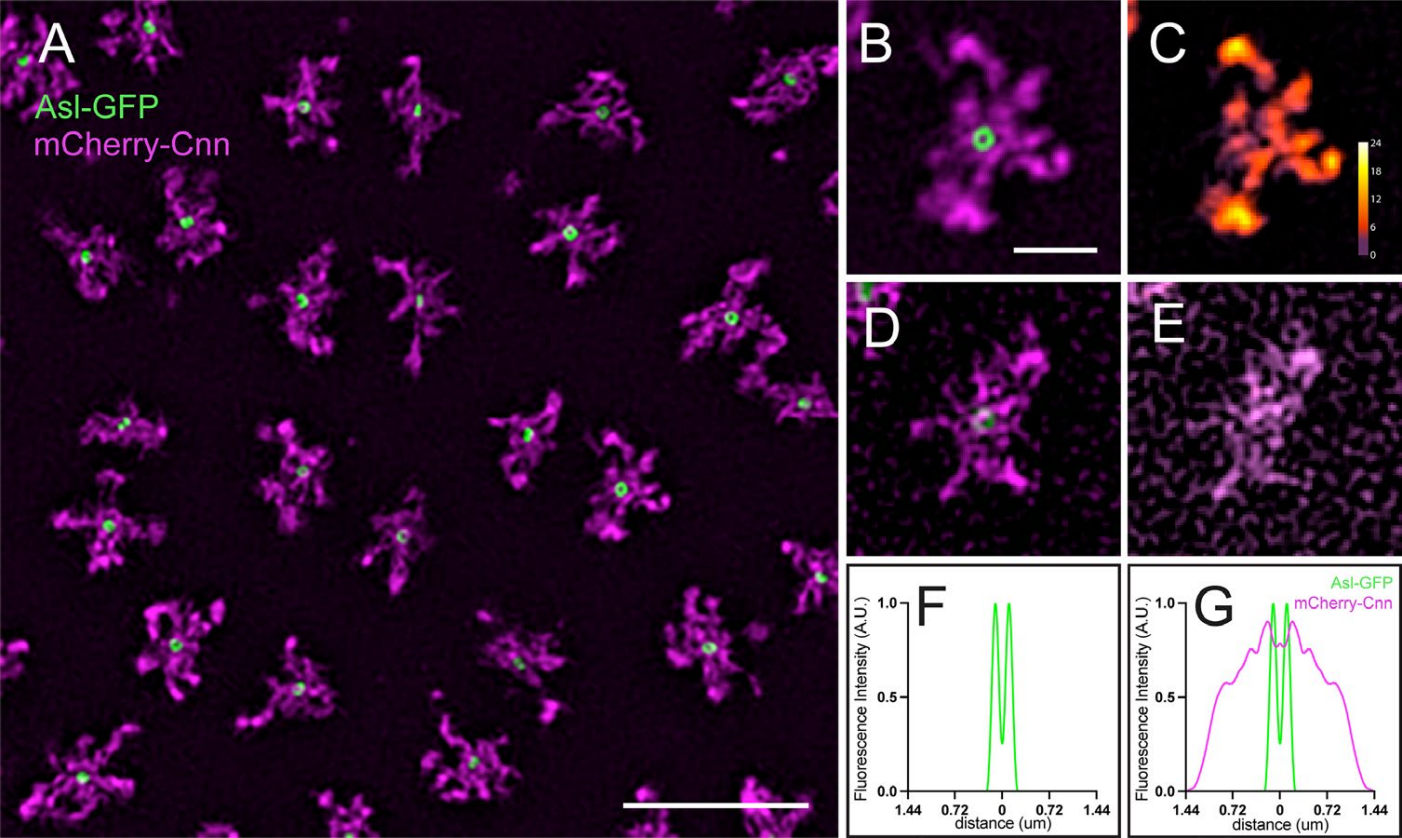
Combining radial profiling with three-dimensional structured illumination microscopy (3D-SIM). (A–E) 3D-SIM images of centrosomes labelled with mCherry-Cnn [pericentriolar material (PCM)] and Asl-GFP (centrioles). The centriole Asl signal is resolved as a ring with 3D-SIM but not confocal imaging (see Figure 3A for comparison). Example centrosomes imaged with (B) optimal or (D) sub-optimal imaging conditions. Modulation contrast-to-noise ratio (MCNR) maps showing well-reconstructed centrosomes (C) vs. poorly reconstructed structures (E). Light colors (white/yellow/orange) indicate reliable reconstruction; dark colors (purple/red) indicate artefacts. Scale bar: 5 μm (A) and 1 μm (B–E). (F) Radial profile of Asl-GFP or (G) Asl-GFP and mCherry-Cnn.

Among available super-resolution techniques, 3D-SIM is particularly useful for live samples because it requires relatively low illumination intensity. Here, I describe how 3D-SIM is performed (example shown in Figure 4A).

1. Collect and prepare the embryos as described in section A. Place the MatTek dish on the microscope. A 60× 1.42 NA objective mounted on a SIM microscope fitted with 488 nm (~800 μW at sample) and 593 nm (~170 μW at sample) laser lines is typically used.

2. Apply immersion oil with a refractive index of 1.514 to the objective. This refractive index is optimal for GFP/mCherry when centrosomes are localized at the cortex. If using an objective with a correction collar, adjust the collar to maximize the centrosome fluorescence intensity. (At the beginning of the experiment, iteratively adjust the collar to give the highest fluorescence values at the centrosome at a given laser power and exposure time.)

3. Set the z-stack to 0.6 μm total height with 0.125 μm step size to minimize unnecessary photobleaching.

4. Set camera exposure times as short as possible while maintaining sufficient contrast to enable accurate reconstruction of the centrosome signal.

5. Acquire images.

6. Define reconstruction parameters using an empirically determined optical transfer function (OTF) optimized for fluorophore and refractive index with the SIMcheck plugin in FIJI/ImageJ [35].

7. Set the Weiner filter to 0.006. The optimum value can be assessed using SIMcheck.

8. Perform reconstruction using the software provided with the microscope.

*Note: In the example shown here, I use a fly stock where the protein of interest is tagged with mCherry and a general centrosomal marker is tagged with eGFP. 3D-SIM acquisition requires multiple raw images (15 per reconstructed frame). As mCherry is relatively photounstable, and the 488 nm laser readily bleaches mCherry, the red channel must be acquired first.*

9. Quality control is critical for super-resolution imaging, as reconstruction algorithms can generate artefacts. It is essential to confirm that reconstructed features correspond to structures genuinely present in the biological sample, rather than artefacts introduced during image reconstruction (see Figure 4B–E for examples).

10. Open the raw and reconstructed images in ImageJ.

11. Using SIMcheck, assess the modulation contrast to noise ratio by choosing the option *Modulation contrast on the raw data*. This compares the intensity of the illumination stripe pattern with the underlying signal.

12. SIMcheck generates a color-coded map where high contrast to noise modulation is shown as a light color (white/yellow/orange; see example in Figure 4B, C). Only centrosomes located within these regions should be used for further analysis. Structures in regions with low modulation contrast-to-noise cannot be trusted, as they may represent artefacts (see Figure 4D, E).

13. Analyze SIM images as described in section C. In Figure 4F, G, profiles are normalized so that the peak intensity is set to 1, giving localization at a higher resolution.

## Validation of protocol

This protocol has been validated in the following research articles:

• Conduit et al. [8]. A molecular mechanism of mitotic centrosome assembly in *Drosophila. eLife* 3 (Figures 1 and 4).

• Hu et al. [28]. The conserved Spd-2/CEP192 domain adopts a unique protein fold to promote centrosome scaffold assembly. *Sci Advances* 11 (Figures 2, 3, and 7–9).

## General notes and troubleshooting

1. Heptane glue loses adhesiveness over time; prepare fresh for optimal embryo mounting.

2. Voltalef oil prevents desiccation and enables embryo development during imaging. If the embryo dechorionation has taken too long, the cortex will take on a wavy appearance on the microscope.

3. In FRAP experiments, bleaching an ROI that is too large will cause cytoplasmic signal loss, and the recovery seen may be misleading.

4. Fluorophore colors can be swapped in FRAP experiments: in this context, the GFP signal should ideally be bleached using a 440 nm laser, as a 488 nm laser will also bleach the mCherry signal.
